# Molecular Subtype Classification and Mechanistic Investigation Based on Ferroptosis‐Related lncRNAs in Ovarian Cancer

**DOI:** 10.1155/genr/4503115

**Published:** 2026-03-20

**Authors:** Qianru Li, Mengyao Lv, Aijie Zhang, Xue Liu, Wanting Lu, Min Shao, Limian Cao

**Affiliations:** ^1^ Department of Critical Care Medicine, The First Affiliated Hospital of Anhui Medical University, Hefei, Anhui, 230001, China, ahmu.edu.cn; ^2^ Department of Immunology, School of Basic Medical Sciences, Anhui Medical University, Hefei, Anhui, 230032, China, ahmu.edu.cn

**Keywords:** AC027348.1, ferroptosis, lncRNA, molecular subtypes, ovarian cancer

## Abstract

Ovarian cancer (OC) ranks among the most prevalent malignant tumors in women, contributing significantly to mortality rates. This disease exhibits considerable heterogeneity, characterized by intricate molecular and genetic alterations. Ferroptosis, a distinct form of cell death, has emerged as a critical factor in various cancers, including OC. However, the regulatory mechanisms underlying ferroptosis in OC patients remain unclear and require further investigation. This study aimed to identify lncRNA associated with OC and elucidate the underlying mechanisms through bioinformatics methods and experimental validation. Ferroptosis‐related lncRNAs (FRLs) was identified in OC, and its prognostic value was assessed using univariate Cox analysis. Additionally, the molecular subtypes of FRLs were evaluated through the ConsensusClusterPlus software package. Notably, Cluster 2 exhibited a low TME score, which was linked to a poorer prognosis. The GSEA suggested that Cluster 2 shared similarities with other clusters associated with worse survival outcomes, likely due to the activation of tumor‐associated pathways. In vitro experiments further confirmed that certain lncRNAs could be upregulated under ferroptotic conditions. We focused on two lncRNAs, AC027348.1 and TRAM2‐AS1, which were not noticed before and were significantly upregulated, to explore their regulatory effects on ferroptosis. The underlying mechanisms were preliminarily investigated through transcriptome sequencing. In summary, our findings offer new insights into the pathogenesis of OC, particularly regarding the role of lncRNAs related to ferroptosis.

## 1. Introduction

Ovarian cancer (OC) is one of the most prevalent cancers among women and a leading cause of mortality due to gynecologic malignancies [1]. According to the latest global cancer statistics from 2020, there were 313,959 new cases of OC and approximately 207,000 deaths, ranking it as the eighth leading cause of cancer‐related deaths in women [2]. The high mortality rate is attributed to tumor heterogeneity, the absence of reliable early diagnostic methods, and a significant incidence of chemotherapy‐resistant recurrences. Despite advancements in chemotherapy and surgical interventions, the 5‐year survival rate for patients with advanced OC remains low [3, 4]. In fact, while the 10‐year survival rate for OC patients is reported at 55%, it drops to a mere 15% for those with advanced disease. Consequently, effective diagnostic markers and therapeutics are identified [5].

Strategies are essential to enhance the prognosis for patients with OC. Ferroptosis is a distinct form of iron‐dependent programmed cell death that differs significantly from necrosis, apoptosis, and autophagy at the morphological, biochemical, and genetic levels [6, 7]. This process leads to cell damage and death through iron‐dependent lipid peroxidation [8–10]. Key manifestations of ferroptosis include changes in mitochondrial morphology and integrity, such as the reduction in organelle size, diminished mitochondrial cristae, loss of mitochondrial membrane density, and rupture of the outer mitochondrial membrane [11–13]. Recently, ferroptosis‐related genes have been identified as potential diagnostic markers, and the induction of ferroptosis in cancer has emerged as a promising therapeutic strategy, particularly for drug‐resistant cancers [14]. However, in OC, only a limited number of ferroptosis‐associated therapeutic targets have been recognized.

Long noncoding RNA (lncRNA), which are noncoding RNAs longer than 200 nucleotides, have been widely studied for their roles in tumor proliferation, metastasis, cell cycle regulation, and programmed cell death. Although lncRNAs are primarily not translated into proteins, they are crucial in tumorigenesis [15–17]. For instance, Wu et al. demonstrated that lncRNA NEAT1 regulates the sensitivity of lung cancer to ferroptosis [18]. Additionally, Zhang et al. showed that lncRNA HEPFAL promotes ferroptosis in hepatocellular carcinoma by modulating the ubiquitination of SLC7A11 [19]. Furthermore, lncRNA MALAT1 knockdown has been found to enhance Erastin‐induced ferroptosis in endometriosis [20]. In the context of OC, ferroptosis‐related lncRNAs (FRLs), such as lncRNA CACNA1G‐AS1, can encourage the malignant phenotype of OC cells by influencing iron death through the FTH1‐IGF2BP1 axis [21]. Moreover, ADAMTS9‐AS1 has been shown to mitigate ferroptosis by targeting the miR‐587/SLC7A11 axis in epithelial OC [22]. However, the number of identified FRLs in OC patients remains small, highlighting the need for further research to discover novel lncRNAs as potential therapeutic targets against OC.

The objective of this study was to investigate FRLs (particularly AC027348.1, which will be mentioned later) in OC and evaluate their prognostic significance. We obtained expression and survival data for TCGA‐OC patients from the UCSC XENA database. These data were utilized to identify FRLs with prognostic value in OC, establish molecular subtypes associated with OC, and analyze differences in prognosis, clinical features, pathway enrichment, and tumor microenvironment (TME) among the subtypes. Therefore, we hypothesize that lncRNA AC027348.1 and TRAM2‐AS1 exert regulatory effects on ferroptosis. We intend to conduct preliminary in vitro experiments to investigate the underlying regulatory mechanisms of AC027348.1. Overall, this research offers new insights into the relationship between FRLs and OC pathogenesis.

## 2. Materials and Methods

### 2.1. Datasets and Sample Extraction

TCGA‐OC patient sequencing, survival data, and level 3 HTSeq‐fragments per kilobase million (FPKM) data were obtained from the UCSC Xena website. Samples without survival data were excluded, resulting in a final dataset of 376 samples. For the sequencing data, the UCSC Xena platform employed a log2 transformation (log2 (FPKM + 1)), which normalized the expression data and ensured it followed a distribution consistent with normality. Additionally, Ensembl IDs within the expression matrix were mapped to gene symbols using gencode.v22.annotation.gene.probeMap. From this annotation, the types of gene symbols were determined, leading to the identification of 14,068 lncRNAs.

### 2.2. Ferroptosis‐Related Gene Detection

A total of 123 ferroptosis‐related marker genes (mRNA) were sourced from FerrDb (https://www.zhounan.org/ferrdb) (Supporting Table S1), a dedicated database that compiles genes associated with ferroptosis and provides relevant information about markers, related molecules, and associated diseases. Utilizing the mRNA expression data from TCGA‐OC, 95 ferroptosis‐related marker genes were successfully identified.

### 2.3. Identification of the Ferroptosis‐Related lncRNA Prognostic Signature

Pearson correlation analysis was conducted to explore the relationship between ferroptosis‐related marker genes and lncRNAs, considering associations significant if the correlation coefficient met the criteria |R| > 0.3 and *p* < 0.001*.* This analysis resulted in the identification of 1388 genes (Supporting Table S2). Furthermore, univariate Cox regression analysis revealed 65 lncRNAs whose expression levels were significantly associated with patient outcomes, highlighting their potential prognostic value for OC (*p* < 0.01, Supporting Table S3).

### 2.4. Consensus Clustering Analysis Based on FRLs

Cluster analysis was executed using the “ConsensusClusterPlus” package to uncover novel molecular subtypes [23]. The analysis identified various new subtypes through the consensus cumulative distribution function (CDF) plot, delta area plot, and cluster‐consensus synthesis. The log‐rank test for these subtypes was conducted using the survival and survminerr packages, with results visualized as KM curves. Furthermore, the Kruskal–Wallis rank sum test was employed to evaluate differences in prognosis‐related lncRNAs across the three identified subtypes, with findings represented in heatmaps. Principal component analysis (PCA) plots were generated using the R package scatterplot3D. Notably, microenvironmental scores exhibited variations across different subgroups, a difference that was statistically confirmed by the Kruskal test.

### 2.5. Functional Enrichment Analysis of Clusters and Risk Groups

The enrichment landscapes from seven databases (KEGG, REACTOME, WikiPathWays, GO, CancerSEA, HallMark) were assessed using a comprehensive GSEA strategy, focusing on three isoforms.

### 2.6. Cell Culture

The OVCAR8 cell line was obtained from Dr. Bo Chu at the Department of Cell Biology, School of Basic Medical Sciences, Cheeloo College of Medicine, Shandong University. The OVCA433 cell line was purchased from Huiying Biological Technology Co. Ltd. (Shanghai, China. Product catalog number: ALWS‐1142). The SKOV3 (Serial: SCSP‐5214) and HEK293T (Serial: SCSP‐5209) cell lines were acquired from the Cell Bank of the Chinese Academy of Sciences (Shanghai, China). OVCAR8 cells were cultured in Dulbecco’s modified Eagle’s medium (DMEM, Gibco), while OVCAR433 and SKOV3 cells were cultured in RPMI‐1640 medium. All culture media were supplemented with 10% fetal bovine serum (Biological Industries) and 1% penicillin–streptomycin (Gibco). Cells were incubated at 37°C in a 5% CO_2_ incubator (Thermo Scientific).

### 2.7. Plasmids and Cloning

Full‐length sequences of long noncoding RNAs were obtained from the NCBI and UCSC databases. These sequences were then uploaded to the short hairpin RNAs (shRNAs) design website (https://rnaidesigner.thermofisher.com/), and the siRNA Wizard (online tool) was used to select and design shRNA (performed by InvivoGen). The shRNA knockdown sequences for the target genes were also designed. The Nanjing Prime Biotechnology Co synthesized the oligo primers, which were then annealed and ligated into the digested pLK0.1 vector. HEK293T cells were cotransfected with the vector in Opti‐MEM (Gibco) using the lentiviral packaging reagent LentiFitTM. The primers and their corresponding sequences used in molecular cloning are listed in Supporting Table S4.

### 2.8. Cell Viability Assay

A single‐cell suspension containing 3000 cells per well was plated in 96‐well plates. After allowing the cells to adhere, they were treated with the ferroptosis inducer RSL3 for a duration of 4–12 h. Upon observing the characteristic phenotypes of ferroptosis, the culture medium was replaced with media containing 10% CCK8 reagent. After a 1‐h incubation, the optical density (OD) was measured at 450 nm using an enzyme marker (Thermo Scientific).

### 2.9. Cell Death Assay

Cells were digested to create a single‐cell suspension and then cultured in 24‐well plates. Once the plates reached 80%–90% confluency, the cells were treated with RSL3 and the ferroptosis inhibitor ferrostatin‐1 (Fer‐1) for 4–12 h. Upon observing ferroptosis phenotypes, SYTOX Green Nucleic Acid Dye (Invitrogen), at a ratio of 1: 30,000, was added to plates and incubated in a 5% CO_2_ atmosphere at 37°C for 15 min. Images were captured with an inverted fluorescence microscope (Olympus, Japan) from at least three random fields of view. The images were processed using ImageJ software, and the green‐stained cells were counted as dead cells to determine the ratio of dead to living cells.

### 2.10. Lipid Peroxidation Analysis

The single‐cell suspension was cultured in 6‐well plates, and ferroptosis inducers and inhibitors were introduced once the cell density reached 80%–90%. Afterward, the cells were collected, washed with PBS, and incubated with 5 μM BODIPY‐C11 581/591 (Thermo Scientific) for 30 min at 37°C. The cells were then washed twice more with PBS and resuspended in 300 μL of PBS. The levels of lipid ROS were analyzed using a Becton Dickinson FACS Calibur machine with the FL1 channel, and the data were processed using FlowJo. For each sample, 10,000 cells were analyzed.

### 2.11. Nuclear Plasmid Separation

The cell precipitate was collected from the Petri dish and combined with 300 μL of lysed cytoplasmic buffer, 1 μL of RNAase inhibitor, and 100 m MPMSF (1:100). The mixture was immediately placed on ice for a 20‐min lysis period. Following this, the cells were mixed with precooled 10% NP‐40 solution for 2 min and then centrifuged at 4°C at 5000 rpm for 5 min. Approximately 200 μL of the cytoplasmic supernatant was transferred to a new enzyme‐free EP tube and treated with 300 μL of TRIzol for RNA extraction. Subsequently, 500 μL of PBS was added to the precipitate, which was washed twice and centrifuged again at 4°C at 5000 rpm for 5 min to collect the precipitates. To this, 200 μL of PBS was added to create a homogeneous solution, followed by treatment with 300 μL of TRIzol to extract cytoplasmic RNA. Finally, the nucleoplasmic localization was assessed by RT‐qPCR, using actin and U1 as internal references for cytoplasmic and cytosolic controls, respectively.

### 2.12. Real‐Time RT‐qPCR

Total RNA extraction was performed using TRIzol (Themo). Complementary DNA was synthesized with oligo(dT) and random primers, with primers synthesized by Nanjing Prime Biotechnology Co. GAPDH served as the internal control. The sequences of the primers utilized in this study are listed in Supporting Table S4.

### 2.13. Transcriptome Analysis

The wild‐type (WT) OVCAR8 cell line served as the control group, while the AC027348.1 knockdown groups were established separately. Each group consisted of three replicates, resulting in a total of 12 sequencing samples. To minimize human errors during the sample collection process, all samples were collected by a single operator. Subsequently, the samples were dispatched to Shenzhen Huada Genetics Co. for RNA‐seq sequencing analysis. For the quantitative gene expression assessment, a library was constructed and sequenced online. The software DESeq2 was employed to identify differentially expressed genes (DEGs) before and after AC027348.1 knockdown. The criteria for identifying DEGs included a Q value ≤ 0.05 or an FDR ≤ 0.001. A comprehensive analysis of gene functions related to phenotypic changes was conducted using Phyper to evaluate the GO (https://www.geneontology.org/) and KEGG (https://www.kegg jp/) enrichment of DEGs based on the hypergeometric test for enrichment analyses. Q value ≤ 0.05 indicated significant enrichment among the candidate genes.

### 2.14. Statistical Analysis

To compare multiple groups, the Kruskal and Wilcoxon tests were utilized, while the Pearson correlation coefficient was employed to assess correlations. Additionally, Kaplan–Meier (K–M) analysis was conducted to plot survival curves. One‐way and multifactorial Cox analyses were performed to calculate 95% confidence intervals (CIs) and hazard ratios (HRs). Statistical analyses were executed using R (4.1.3) software, with a significance threshold of *p* < 0.05. The imaging results from WB and other analyses were repeated three times. Statistical differences among the experimental groups were assessed using GraphPad Prism9. Statistical significance was determined via Student’s *t*‐test with 95% CI. Subgroup analyses were conducted using two‐way analysis of variance (ANOVA) with three repetitions. A *p* value < 0.05 was considered significant, where ∗ indicates *p* < 0.05, ∗∗ indicates *p* < 0.01, and ∗∗∗ indicates *p* < 0.001.

## 3. Result

### 3.1. Identification of Ferroptosis Marker‐Related lncRNAs With Prognostic Value in OC

First, the FPKM expression data for OC patients from TCGA‐OV was downloaded from UCSC Xena, resulting in the identification of 14,068 lncRNAs. Subsequently, ferroptosis‐related marker genes (mRNAs) were identified using the FerrDb website (Supporting Table S1) and screened alongside the FPKM expression data from TCGA‐OV, leading to the discovery of 95 genes. To identify FRLs, the Pearson correlation analysis was conducted, with genes exhibiting correlation coefficients greater than 0.3 considered significant. This analysis yielded a total of 1388 significant genes (Supporting Table S2). A Sankey plot (Figure 1(a)) was created to illustrate the relationships between FRLs and mRNAs. Additionally, univariate Cox regression analyses identified 65 FRLs that were associated with patient prognosis (Figure 1(b), Supporting Table S3). Among these, AC009413.2, AC021016.1, and AC011445.1 emerged as poor prognostic factors (HR > 1), while the remaining 62 lncRNAs were associated with favorable prognostic outcomes.

FIGURE 1Identification of ferroptosis‐related lncRNAs marker with important prognostic value for OC. (a) Sankey plot indicating associations between ferroptosis‐related lncRNAs and ferroptosis marker genes. (b) Forest plot showing HR (95% CI) and *p* *-values* (both *p* < 0.01) for lncRNAs identified using univariate Cox proportional hazards analysis. A total of 62 lncRNAs indicated a good prognosis, while 3 indicated a poor prognosis.

**Figure:**
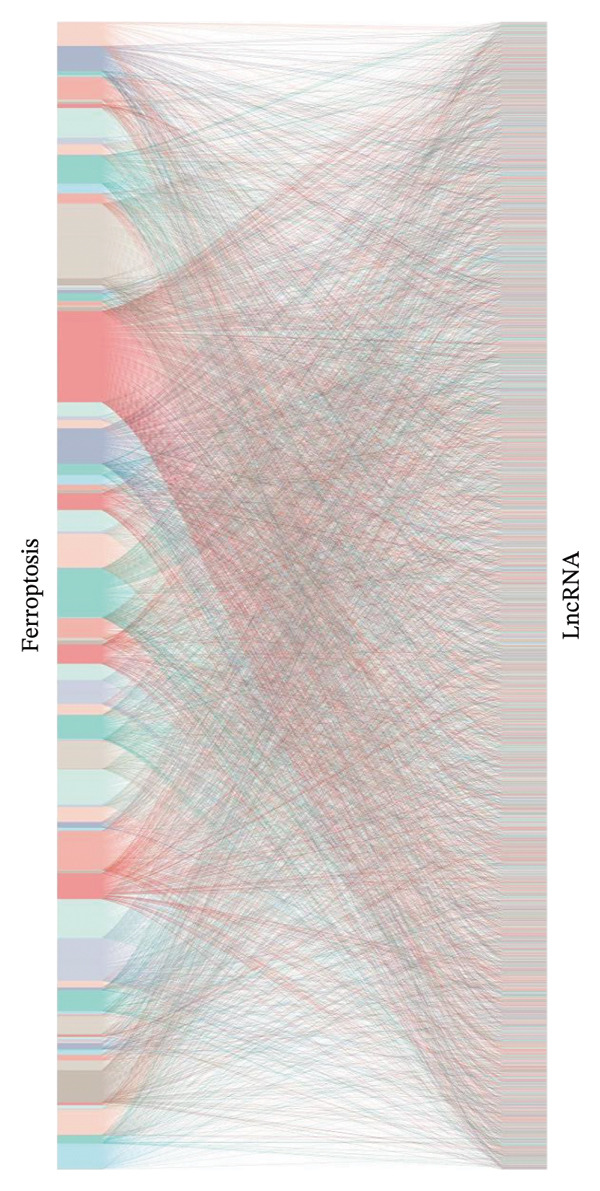
(a)

**Figure:**
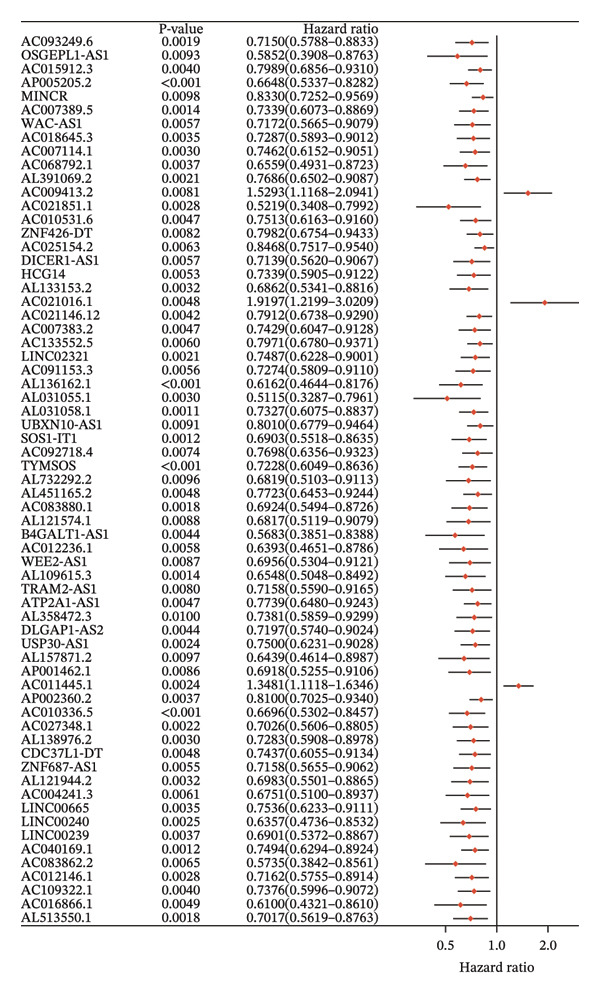
(b)

### 3.2. Identification of Ferroptosis‐Related Subtypes

Clustering analysis was conducted using 65 FRLs to evaluate distinct ferroptosis‐related subtypes in OC patients. The optimal clustering solution identified three unique subtypes, demonstrating intra‐subgroup consistency and stability (Figures 2(a), 2(b), 2(c), 2(d)). A log‐rank test was employed to compare survival across the three subtypes, and Kaplan–Meier curves were generated to visualize these survival differences. Notably, cluster 2 exhibited the poorest survival rates (Figure 2(e)). Furthermore, bar graphs illustrated significant differences in survival outcomes between the three subgroups, underscoring the unfavorable prognosis associated with cluster 2 (Figure 2(f)). A heatmap was created to display variations in gene expression among the three clusters (Figure 2(g)). Furthermore, both 2D and 3D principal component analyses were performed using prognosis‐related lncRNA expression data, highlighting distinct expression patterns across clusters 1–3, thereby reinforcing the robustness of the model (Figure 2(h)).

FIGURE 2Consensus clustering analysis based on FRLs. (a) Consensus clustering matrix at *k* = 3. (b) Consensus clustering CDF for k values between 2 and 9. (c) Relative change in area under the CDF curve when *k* = 3. (d) Cluster‐consensus plot showing the cluster‐consensus value (mean of pairwise consensus values of the members of that cluster) for each classification for different k values. Higher values represent higher stability and vice versa. (e) KM curves of survival differences between C1, C2, and C3 subtypes were significant, as indicated by the log‐rank test. (f) Chi‐squared test for survival differences between C1, C2, and C3 subtypes. (g) Heatmap demonstrates the difference in prognosis‐related lncRNAs among the three subtypes. (h) Principal component analysis (PCA) revealed that cluster 2 had a high‐risk score (shown in blue), while C1 and C3 subtypes had lower risk scores (shown in red and green, respectively) (^∗^ *p* < 0.05, ^∗∗^ *p* < 0.01, ^∗∗∗^ *p* < 0.001, ns, not significant).

**Figure:**
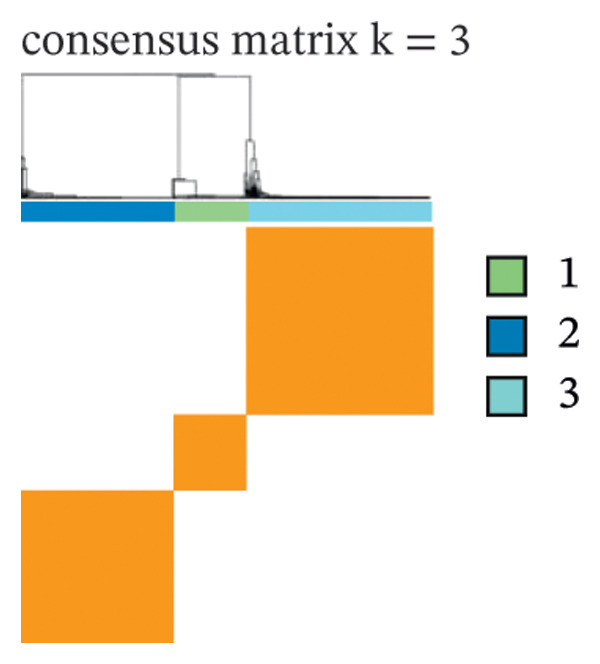
(a)

**Figure:**
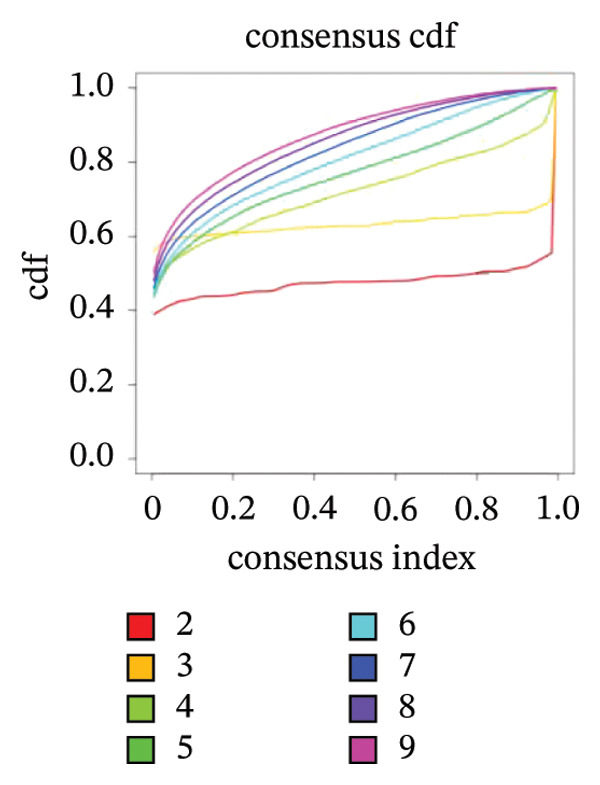
(b)

**Figure:**
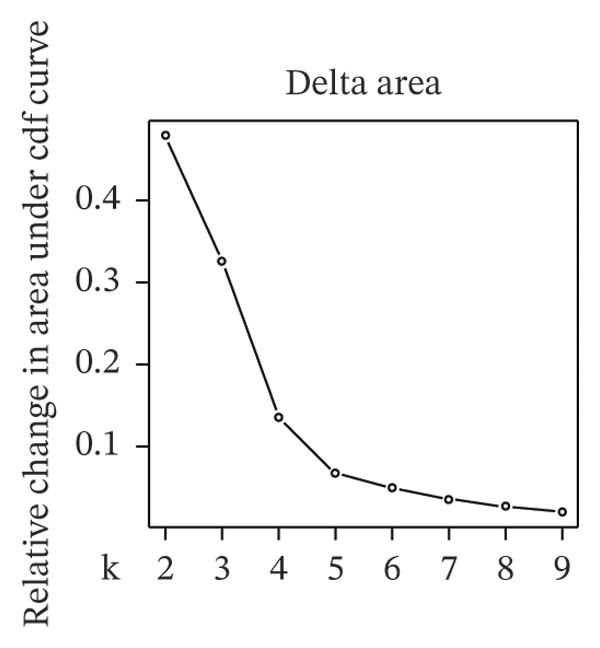
(c)

**Figure:**
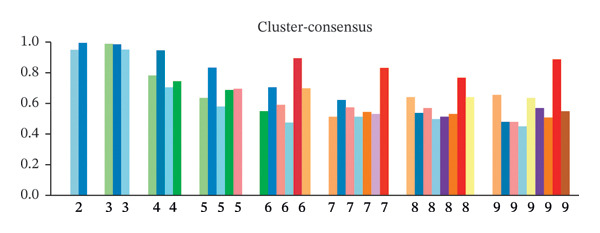
(d)

**Figure:**
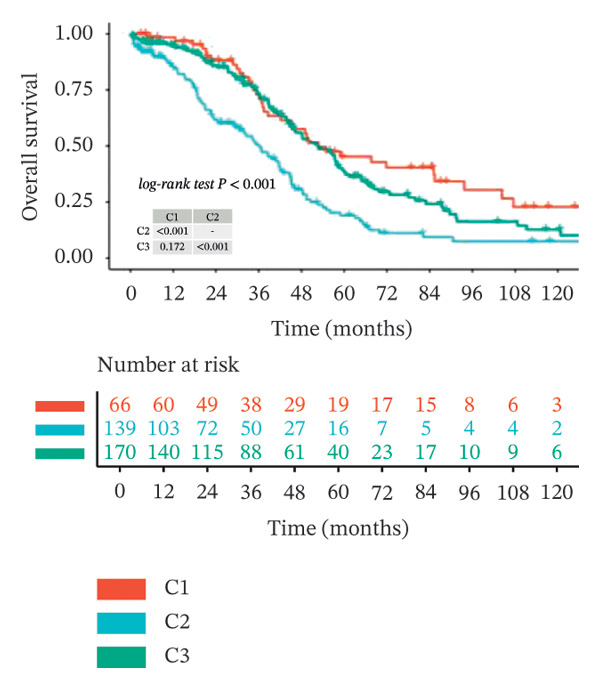
(e)

**Figure:**
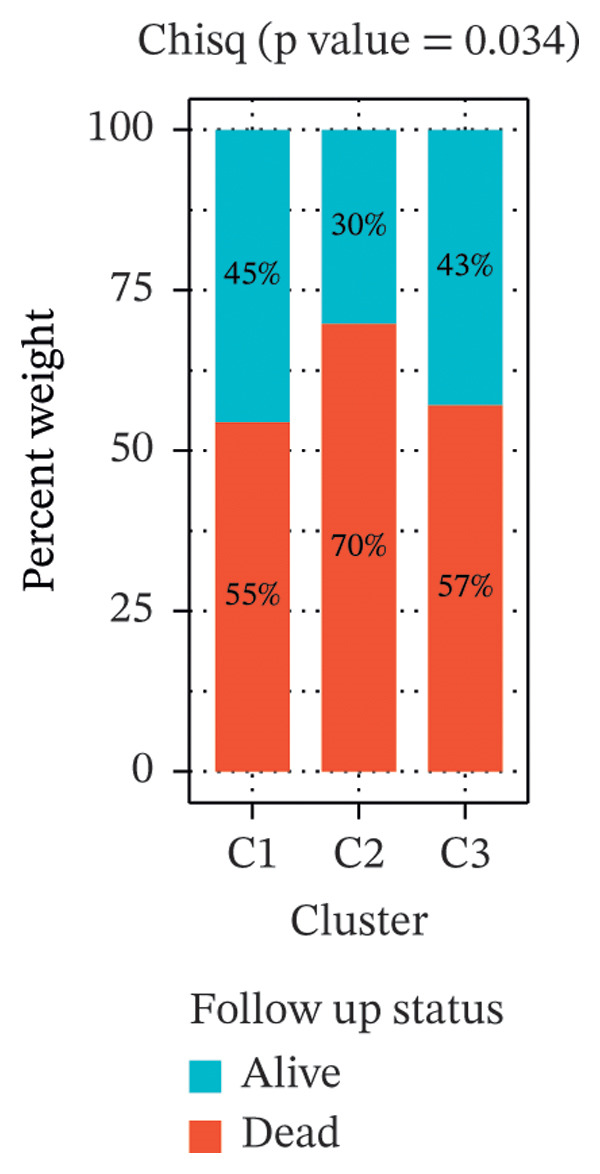
(f)

**Figure:**
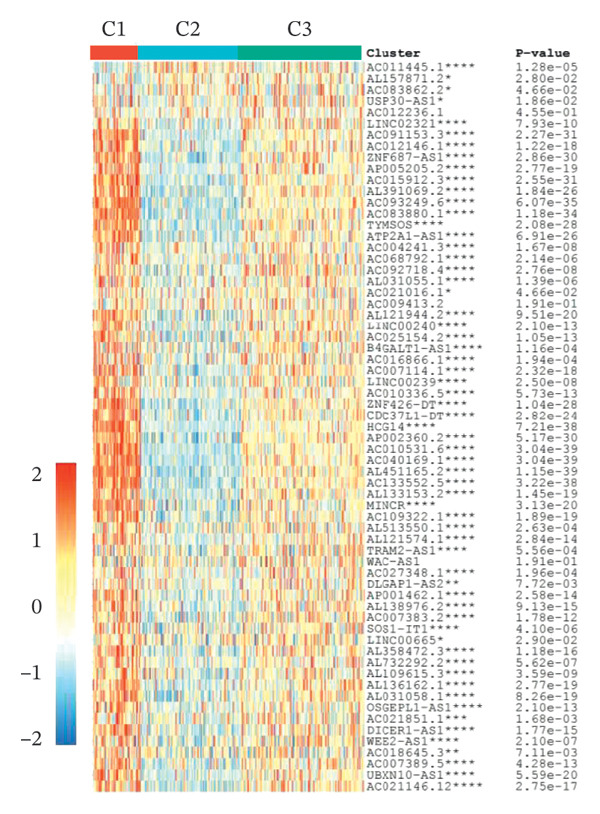
(g)

**Figure:**
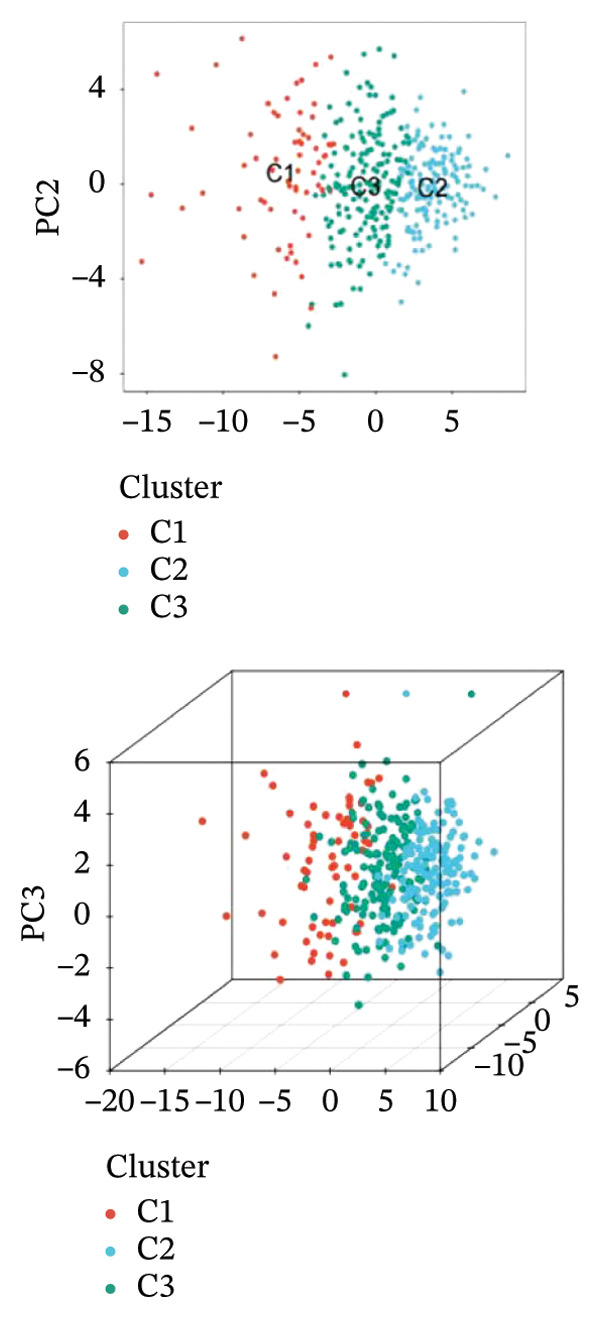
(h)

### 3.3. Correlation Analysis Between Subtypes, Tumor Immune Microenvironment

This is crucial in tumor progression [24]. To explore the association between ferroptosis marker‐related features and antitumor immunity in OC patients, the Kruskal algorithm test was utilized to analyze microenvironment scores across the three subgroups. The results indicated that cluster 2 was linked to lower TME scores and a poorer prognosis (Figure 3(a)). Additionally, this analysis revealed elevated expression of EMT markers, including EMT1, EMT2, and EMT3 and panfibroblast TGF‐β response characteristics (Pan‐F TBRS) in cluster 2. A heatmap visually compared the levels of various immune cells across the three subtypes (Figure 3(b)). Notably, cluster 2 exhibited higher infiltration of immune cell types, including neutrophils, macrophages, plasma cells, and T cells. This suggests that the unfavorable prognosis associated with cluster 2 may be influenced by the TME and the tumor’s immune landscape.

FIGURE 3Correlation analysis between subtype and tumor immune microenvironment: (a) visualization of significant immune cell types as identified by the Kruskal–Wallis rank sum test. CDF: cumulative distribution function, KM: Kaplan–Meier. (b) Kruskal test indicated that the statistical microenvironmental scores differed across subgroups (^∗^ *p* < 0.05, ^∗∗^ *p* < 0.01, ^∗∗∗^ *p* < 0.001, ns, not significant).

**Figure:**
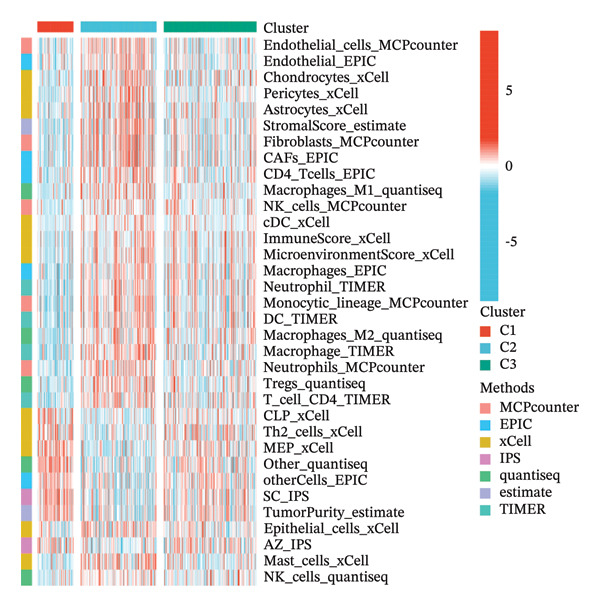
(a)

**Figure:**
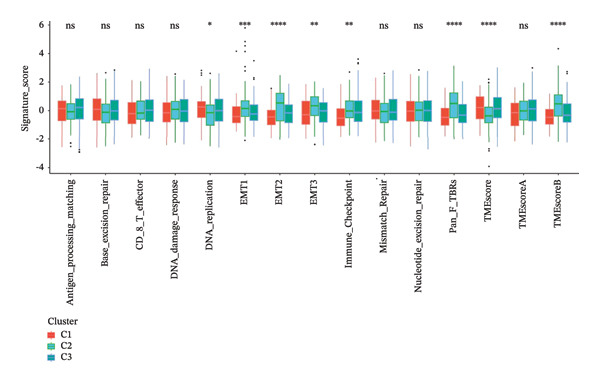
(b)

### 3.4. Discovery of Molecular Functions and Pathway Exploration by GSEA Analysis

**FIGURE 4:**
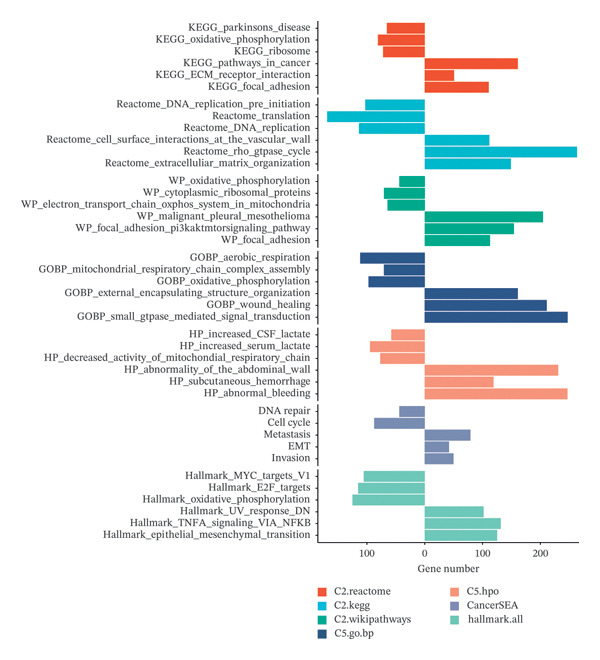
GSEA enrichment analysis. The enrichment landscape of seven databases (KEGG, REACTOME, WikiPathWays, GO, CancerSEA, HallMark) in the C2 subtype was compared with the C1 and C3 subtypes. The right direction of the bar graph indicates that the modified pathway is significantly enriched in the C1, C2, and C3 subtypes, respectively.

A comprehensive GSEA analysis was conducted to evaluate the enrichment profiles of seven gene sets in cluster 2 in comparison to clusters 1 and 3. The top three pathways that exhibited significant enrichment in either cluster 2 or the combined clusters 1 and 3 were selected for visualization (Figure 4, Supporting Table S5). The GO gene ontology analysis indicated that cluster 2 was primarily enriched in processes related to external encapsulating structure organization, wound healing, and small GTPase‐mediated signal transduction, as identified in the REACTOME analysis. Additionally, cluster 2 was closely associated with cell surface interactions at the vascular wall, the Rho GTPase cycle, and the organization of the extracellular matrix. Metabolic pathway analysis conducted via WIKIPATHWAYS revealed that cluster 2 was predominantly linked to malignant pleural mesothelioma, the PI3K/AKT/mTOR signaling pathway, and focal adhesion. Collectively, these findings suggest that cluster 2 is associated with a poorer survival rate compared to the others.

### 3.5. FRLs Affect Erastin/RSL3‐Induced Ferroptosis

To explore the relationship between lncRNA and ferroptosis in OC cells, we performed in vitro experiments to establish the connection between the identified FRLs and the induction of ferroptosis. OVCAR8 cells were treated with RSL3 and Erastin to effectively induce ferroptosis. Following the treatment, we observed significant cellular changes indicative of ferroptosis and subsequently extracted RNA to rigorously analyze the expression alterations of the identified FRLs (Figure 4). The heatmap depicted the fold changes of 12 FRLs and SLC7A11 in OVCAR8 cells post‐treatment with RSL3 and Erastin. Bar graphs illustrated the specific expression changes among these 12 FRLs and SLC7A11 (Figures 5(a) and 5(b)). Notably, AC027348.1, TRAM2‐AS1, PART1‐AS1, POLH‐AS1, AL109615.3, AC021016.1, and SLC7A11 were significantly upregulated in response to RSL3‐induced ferroptosis (Figure 5(c)). Similarly, AC027348.1, TRAM2‐AS1, PART1‐AS1, POLH‐AS1, AL109615.3, AC021016.1, AC007383.1, and SLC7A11 were upregulated during Erastin‐induced ferroptosis, while WAC‐AS1 exhibited downregulation in both RSL3 and Erastin treatments (Figure 5(d)).

FIGURE 5FRLs in OVCAR8 cells are altered in response to ferroptosis (a) Schematic flow of dosing experiments. (b–d) Heatmap of the trend of changes in FRLs in OVCAR8 cells stimulated with the ferroptosis‐inducing drug RSL3 (0.5 μm) for 6 h and Erastin (20 μm) for 24 h, and bar graphs (^∗^ *p* < 0.05, ^∗∗^ *p* < 0.01, ^∗∗∗^ *p* < 0.001, ns, not significant. The bars indicate mean ± SEM values. All data were statistically analyzed using Student’s *t*‐test).

**Figure:**
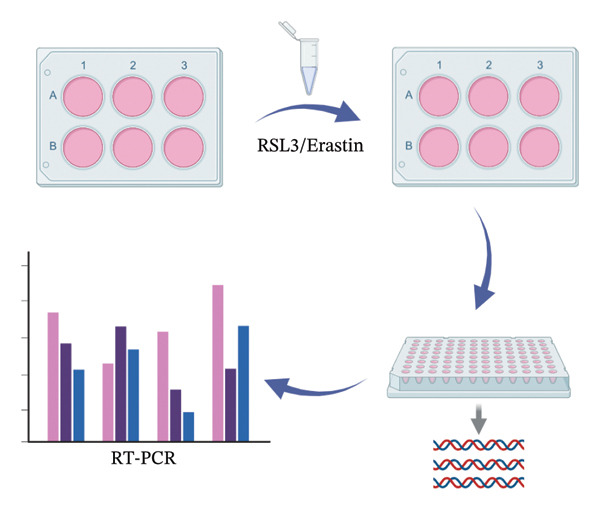
(a)

**Figure:**
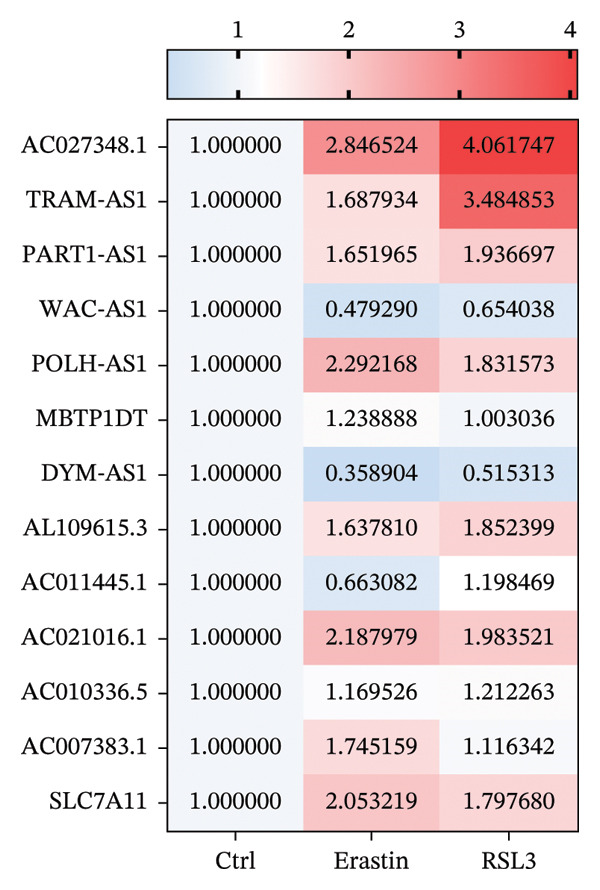
(b)

**Figure:**
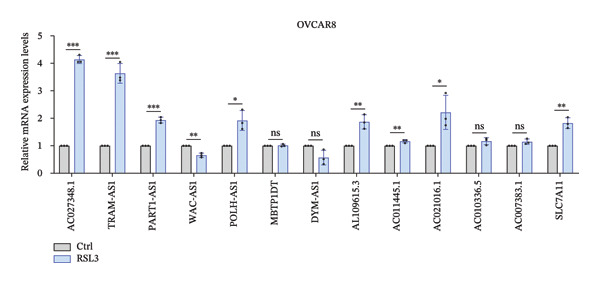
(c)

**Figure:**
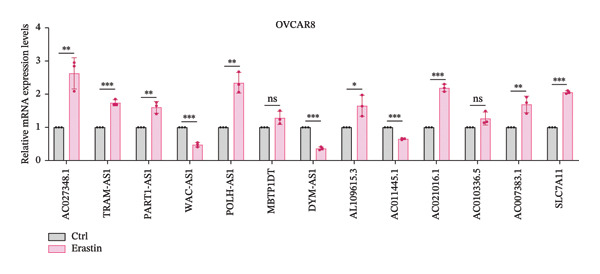
(d)

We further validated the lncRNAs that demonstrated significant expression changes across various dosing concentrations in two additional OC cell lines: SKOV3 and OVCAR433. In the SKOV3 cell line, TRAM2‐AS1 and POLH‐AS1 were significantly upregulated, whereas WAC‐AS1 showed significant downregulation in response to different concentrations of Erastin and RSL3 stimulation (Figures 6(a) and 6(b)). These expression changes were consistent in the OVCAR433 cell line as well (Figures 6(c) and 6(d)). The results of these experiments indicate that ferroptosis in OC cells is associated with elevated levels of certain lncRNA expressions.

FIGURE 6FRLs in SKOV3 and OVCAR433 cells are altered in response to ferroptosis. (a–b) Histogram of the trend of FRLs in SKOV3 cells stimulated by the ferroptosis‐inducing drugs RSL3 (0.5, 1.0,1.5 μm) and Erastin (5, 10, 20 μm). (c–d) Histogram of the trend of FRLs in OVCAR433 cells stimulated with the ferroptosis‐inducing drugs RSL3 (0.5, 0.75, 1.0 μm) and Erastin (5, 10, 20 μm) (^∗^ *p* < 0.05, ^∗∗^ *p* < 0.01, ^∗∗∗^ *p* < 0.001, ns, not significant. The bars indicate mean ± SEM values. All data were statistically analyzed using Student′s *t*‐test).

**Figure:**
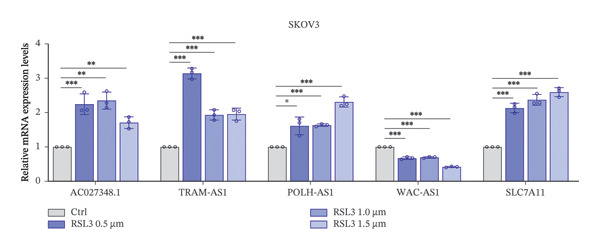
(a)

**Figure:**
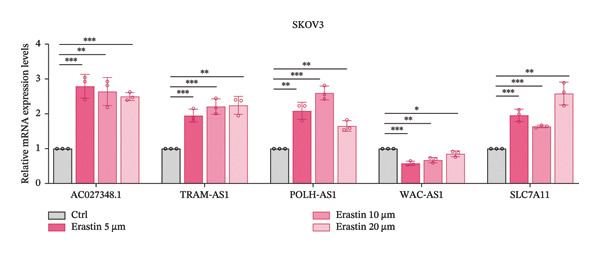
(b)

**Figure:**
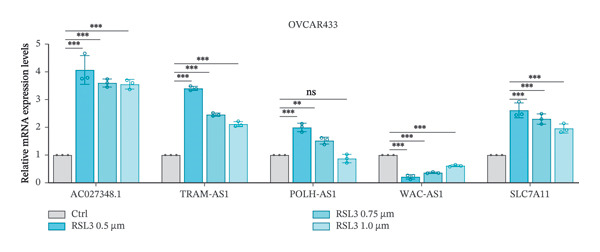
(c)

**Figure:**
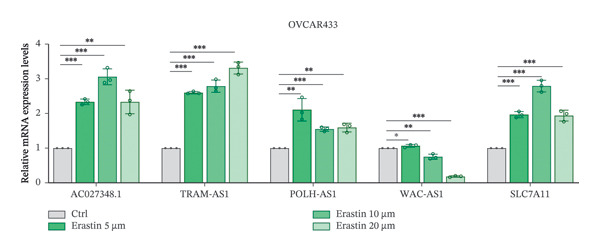
(d)

### 3.6. TRAM2‐AS1 and AC027348.1 Regulate RSL3‐Induced Ferroptosis

Previous studies have established that FRLs are essentially involved in OC pathogenesis. However, the specific mechanisms by which these lncRNAs influence ovarian carcinogenesis through the modulation of ferroptosis remain unclear. In this study, we focused on two lncRNAs, TRAM2‐AS1 and AC027348.1, which exhibited significant changes in expression from our dosing experiments. Our observations indicate that the downregulation of either TRAM2‐AS1 or AC027348.1 (Figures 7(a) and 7(b)) enhances cell resistance to ferroptosis induced by RSL3 (Figures 7(c) and 7(d)). In contrast, the ferroptosis inhibitor Fer‐1 completely mitigates ferroptotic cell death (Figures 7(e) and 7(f)). Moreover, BODIPY‐C11 staining demonstrated increased levels of endogenous lipid peroxidation in sh‐ctrl cells, while sh‐TRAM2‐AS1 and sh‐AC027348.1 cells showed no such elevation (Figures 7(g) and 7(h)). These findings suggest that AC027348.1 and TRAM2‐AS1 play significant roles in regulating ferroptosis.

FIGURE 7Regulation of ferroptosis by lncRNA AC027348.1 and TRAM2‐AS1. The viability of OVCAR8 cells treated with RSL3 for 6 h was assessed after the knockdown of (a–c) AC027348.1 and (b‐d) TRAM2‐AS1 using the CCK8 kit and confirmed by RT‐qPCR. (e‐f) Apoptosis analysis of OVCAR8 treated with RSL3 (500 nm) and Fer‐1 (2 μm) for 3 h. (g‐h) Measurement of lipid peroxidation levels in OVCAR8 treated with RSL3 (500 nm) and Lipro‐1 (2 μm) for 3 h.

**Figure:**
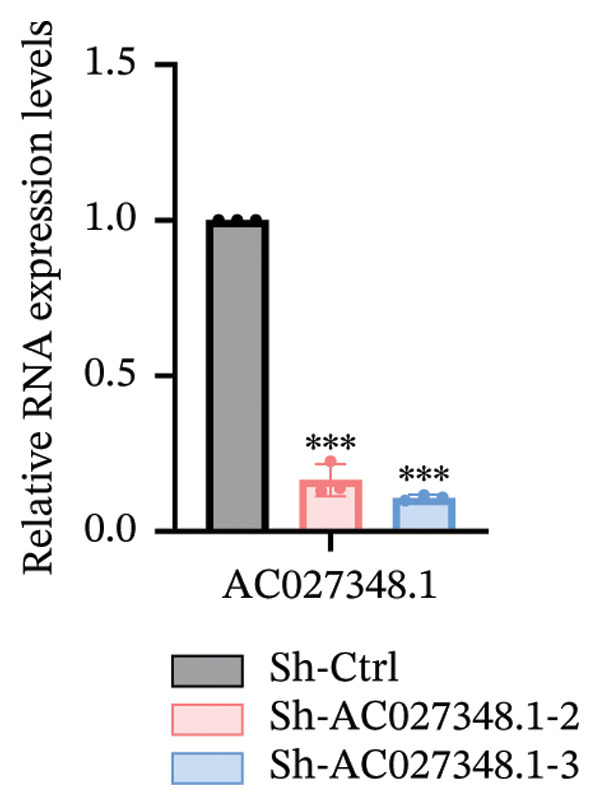
(a)

**Figure:**
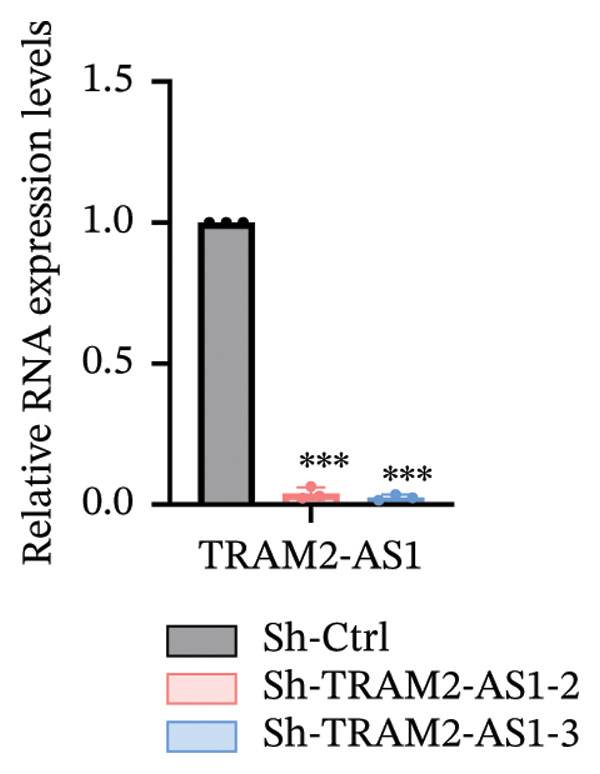
(b)

**Figure:**
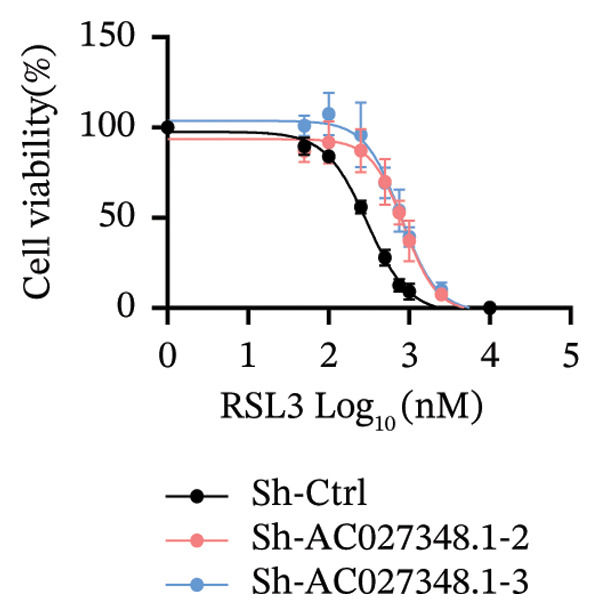
(c)

**Figure:**
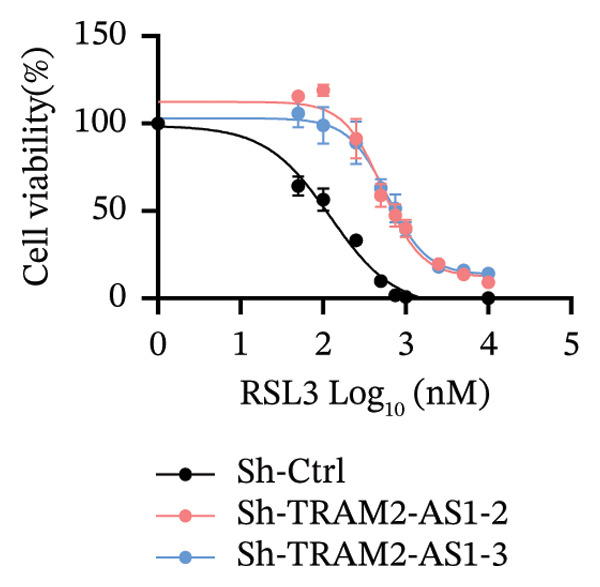
(d)

**Figure:**
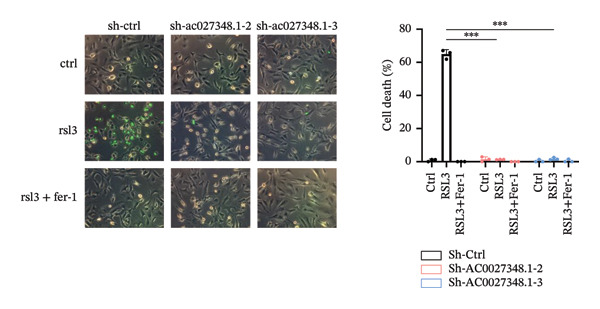
(e)

**Figure:**
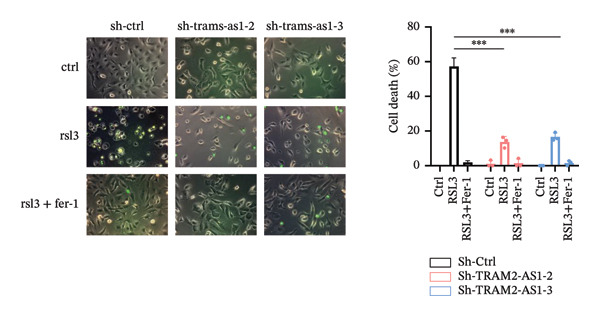
(f)

**Figure:**
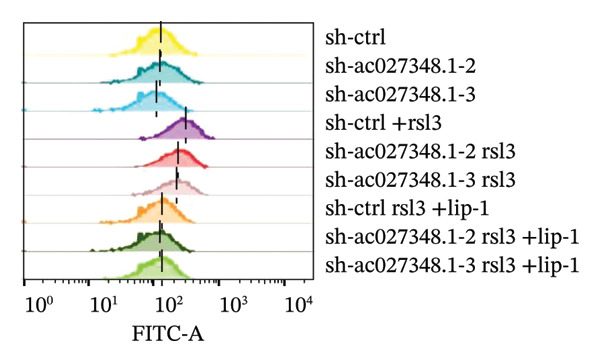
(g)

**Figure:**
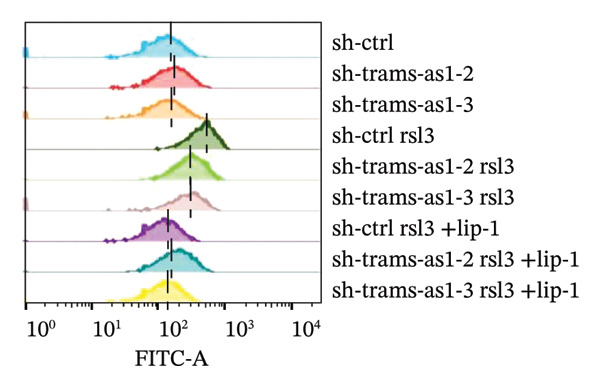
(h)

### 3.7. Preliminary Assessment of AC027348.1 Mechanisms, Which Regulates Ferroptosis

This study aimed to elucidate the mechanisms through which the aforementioned lncRNAs influence ferroptosis. Despite existing literature, there is still a lack of definitive research showing whether lncRNAs directly regulate the drivers of ferroptosis or affect the process via upstream pathways. Therefore, AC027348.1 was employed as the research subject. The gene expression profiles of the control and AC027348.1 knockdown groups were examined using RNA‐seq. As illustrated in Figure 8(a), we identified 6441 RNAs, with 1749 showing increased expression and 1310 demonstrating decreased expression (|log2FC| > 0.5, Q value < 0.05; Supporting Table S6).

FIGURE 8RNA‐seq search for differentially expressed genes. (a) The volcano plot shows 6441 differentially expressed genes detected by RNA‐seq analysis after AC027348.1 knockdown, of which 3059 genes had *p* < 0.05 and |log2FC| > 0.5. Red = gene upregulation, and green = gene downregulation. (b) VENN plot showing the number of shared and unique ferroptosis‐related genes (green) versus the number of differentially expressed genes (blue) identified by RNA‐seq analysis after AC027348.1 knockdown. (c) KEGG enrichment analysis: the *Y*‐axis indicates GO or KEGG entries, and the *X*‐axis indicates the number of enriched genes under the entries. The redder the color and the smaller the *p* *-*value, the more significant the enrichment. The bubble plot *X*‐axis indicates the proportion of genes; the larger the circle, the more genes are present in each pathway. KEGG: Kyoto Encyclopedia of Genes and Genomes, GO: Gene Ontology. (d) Heatmap of the differential expression of genes shared by both the VENN plots.

**Figure:**
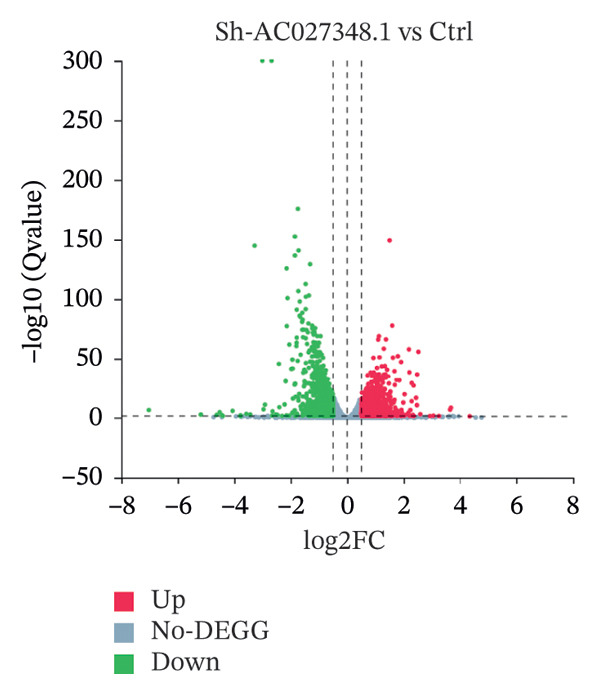
(a)

**Figure:**
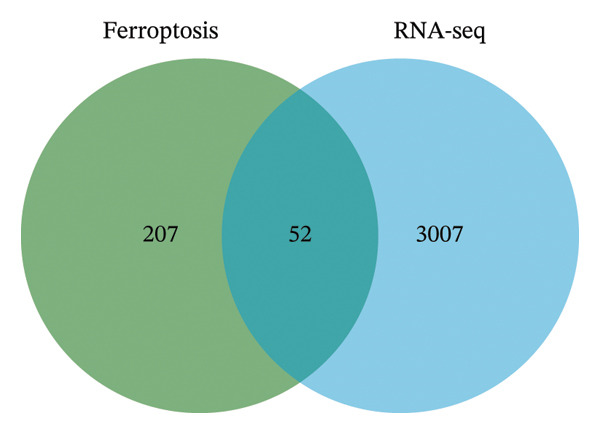
(b)

**Figure:**
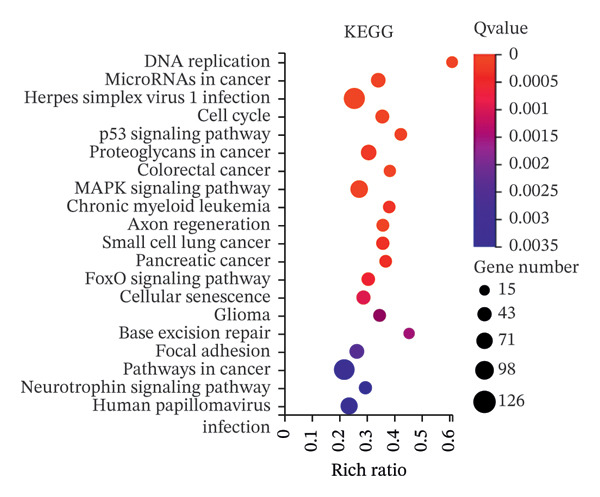
(c)

**Figure:**
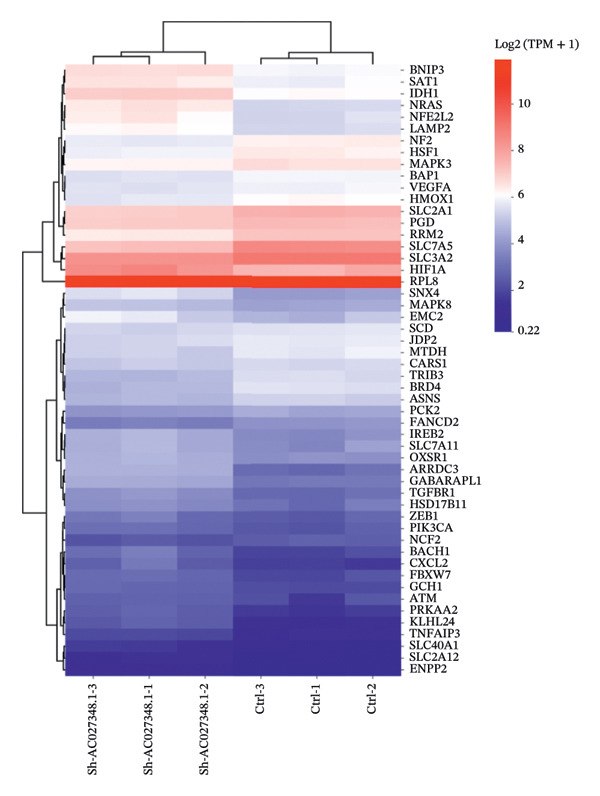
(d)

The GO analysis revealed that the DEGs are predominantly involved in essential biological processes, including DNA replication, cell cycle regulation, cell division, and DNA repair (Supporting Figure 1(a)). The DEGs appear to interact within the cell nucleoplasm, binding to metal ions, DNA, proteins, and ATP (Supporting Figures 1(b) and 1(c); Supporting Table S7). Furthermore, KEGG analysis indicated that these DEGs are primarily associated with pathways related to DNA replication, microRNAs in cancer, and the cell cycle. Additionally, they may play a role in the pathogenesis of herpes simplex virus one infection (Figure 8(c)).

To evaluate the impact of AC027348.1 knockdown on ferroptosis‐related genes, we retrieved a dataset of 259 ferroptosis‐associated genes, encompassing markers, inhibitors, and suppressors, from the FerrDb website (Supporting Table S8). VENN plots illustrated the overlap between DEGs and ferroptosis‐related genes, revealing that 52 genes exhibited altered expression following AC027348.1 knockdown (Figure 8(b)). The clustered heatmap presented in Figure 8(d) highlights the differential expression patterns of these genes. Notable changes were observed in the expression levels of several key genes, including NRF2, SLC40A1, SLC7A5, ARRDC3, and ENPP2. SLC40A1; a critical iron transport protein plays a vital role in maintaining the balance of cellular and systemic iron levels [25]. Additionally, existing literature indicates that SLC40A1 is regulated by miR‐194‐5p and miR‐18a‐5p in ovarian and prostate cancers, respectively. Inhibition of miR‐194‐5p leads to the upregulation of SLC40A1, which in turn reverses cisplatin resistance in OC. Conversely, the inhibition of miR‐18a‐5p promotes SLC40A1 upregulation and hinders the development and progression of prostate cancer [26]. Furthermore, ENPP2, a lipid kinase involved in lipid metabolism, has been linked to the ENPP2/LPA signaling pathway, which plays a crucial role in regulating ferroptosis in cardiomyocytes. The ENPP2/LPA pathway modulates ferroptosis by influencing the expression of GPX4, ACSL4, and NRF2, alongside AKT survival signaling [27]. The mechanisms through which AC027348.1 influences ferroptosis warrant further investigation.

## 4. Discussion

Among gynecologic cancers, OC has the highest mortality rate globally [28]. Recent studies have increasingly demonstrated the significant role of lncRNAs in the development of OC, suggesting their potential as biomarkers for diagnosis and prognosis. For example, the overexpression of the lncRNA XIST, identified as an oncogenic factor in OC, correlates with poor patient prognosis [29]. Additionally, the overexpression of MALAT1 and HOTTIP has been recognized as a critical indicator of OC prognosis [20, 30]. MALAT1 promotes cell proliferation and invasion, and research by Mao et al. suggests that MALAT1 enhances oncogenic activity by inhibiting tumor cell apoptosis, positioning it as a potential diagnostic marker and therapeutic target for EOC. These findings underscore the potential of lncRNAs as prognostic markers for OC. Currently, leveraging the established relationship between lncRNAs and OC, prognostic signatures for autophagy‐associated lncRNAs have been developed [31]. Liang et al. created an immune‐associated lncRNA prognostic model aimed at predicting OC survival, indicating that lncRNAs can serve as both immune and prognostic biomarkers for OC [32]. Additionally, prognostic signatures for FRLs in OC have also been established [33].

Much research has been conducted to advance the understanding of novel molecular subtypes of OC. For example, Zhao et al. employed a consensus clustering approach to identify two distinct clusters characterized by dysregulated copper metabolism [34]. They subsequently applied the Lasso–Cox method to develop a prognostic signature based on DEGs within these clusters. Similarly, Gao et al. identified three cellular pyroptosis gene subtypes, highlighting their potential in predicting prognosis and responses to immunotherapy in OC [35]. Additionally, another study categorized OC patients into three clusters based on the expression patterns of 14 histone acetylation‐related lncRNAs, revealing significant variations in survival probabilities [36]. These findings suggest that such lncRNAs could serve as valuable biomarkers and prognostic indicators for assessing patients’ responses to anticancer treatment. However, there remains a lack of consensus regarding the molecular clustering of FRLs in OC. Identifying the molecular isoforms of FRLs in OC will enhance our understanding of the mechanisms underlying OC progression and treatment.

In the present study, we identified three distinct FRL clusters in OC and investigated their correlations with the tumor immune microenvironment and overall immunity. Our findings suggest that the poor prognosis associated with cluster 2 may be linked to its TME and tumor immune landscape. EMT is known to play a pivotal role in cancer metastasis. Previous research has indicated that activation of the TGF‐β pathway can promote EMT, thereby facilitating the growth and metastasis of ovarian tumors [37]. Our study corroborates these findings, revealing that the EMT and TGF‐β signaling pathways are activated in cluster 2, further establishing its classification as a low‐prognosis subtype. The GSEA enrichment analysis was performed to evaluate the enrichment landscape within cluster 2. The results indicated that cluster 2 shares similar poor survival outcomes with other subgroups, potentially attributable to tumor‐related pathways.

We demonstrated that FRLs can be induced by drugs in three distinct OC cell lines, leading to changes in gene expression. We confirmed the regulatory roles of two significantly upregulated lncRNAs, AC027348.1 and TRAM2‐AS1, in the context of ferroptosis. To explore the mechanisms by which AC027348.1 influences susceptibility to ferroptosis, we employed transcriptomic sequencing. By downloading ferroptosis‐related genes from the FerrDb website and intersecting them with DEGs from our sequencing data, we identified alterations in several genes, including NRF2, SLC40A1, SLC7A5, ARRD3, and ENPP2, following the knockdown of AC027348.1. This suggests that pathways associated with these genes may regulate ferroptosis mediated by AC027348.1. We know that the upregulation of SLC40A1 is related to ferroptosis in OC cells. Therefore, focusing on lncRNAs and SLC40A1 might become a new focus in the research of OC. Additionally, we observed that AC027348.1 is predominantly localized in the nucleus (Supporting Figure 2(a), 2(b), 2(c)), indicating that it may influence the transcription and splicing of relevant pathway genes at the nuclear level.

To further investigate the mechanism by which AC027348.1 modulates ferroptosis, we conducted a series of experimental studies. Given that the pathway involving NRF2 plays a pivotal role in the ferroptosis process, we selected NRF2 as a detection marker. Our experimental results demonstrated that treatment with sh‐AC027348.1 led to a concomitant increase in NRF2 expressions (Supporting Figure 3(a) and 3(b)). However, when cells were treated with sh‐AC027348.1 in an NRF2‐KO cell line, cell viability was significantly reduced, indicating that AC027348.1 can still influence the ferroptosis process independently (Supporting Figure 3(c)). Based on these findings, we hypothesize that AC027348.1 modulates ferroptosis through dual mechanisms: it can act via the RNF2 pathway, as well as through other ferroptosis regulatory factors beyond NRF2. A coanalysis of the DEGs with 259 ferroptosis‐related genes identified 51 genes that had significantly changed expression after AC027348.1 knockdown in NRF2‐KO OC cells. The heat map illustrates the degree of DEGs (Supporting Figure 3(d) and 3(e)). Subsequently, a joint sequencing analysis of the ferroptosis‐related DEGs obtained from WT and NRF2‐KO OVCAR8 cells revealed that 31 genes were differentially changed after knockdown of AC027348.1 in both WT and NRF2‐KO cells (Supporting Figure 3(f)). Circular heatmap analysis of DEGs showed a consistent expression trend in both WT and NRF2‐KO cells; specifically, genes such as KLHC24, SLC40A1, and ARRDC3 were upregulated, whereas SLC7A5 and TRIB3 were downregulated (Supporting Figure 3(g)). Collectively, these data suggest that AC027348.1 exerts independent regulatory control over ferroptosis through multiple pathways, potentially by influencing the NRF2 pathway or by modulating the expression of ferroptosis‐related genes such as SLC40A1, SLC7A5, and ARRDC3.

Nonetheless, a deeper understanding of the specific mechanisms through which long noncoding genes regulate ferroptosis necessitates more extensive cell‐based experiments and clinical trials. This line of research holds promise for expanding the horizons of ferroptosis laboratory studies.

## 5. Conclusion

In conclusion, this study utilized bioinformatics analysis, in vitro experimental validation, and transcriptomic sequencing to identify three subtypes of FRLs associated with OC. Cluster 2 was linked to a poorer prognosis, potentially due to the activation of tumor‐associated pathways. We further confirmed the role of AC027348.1 and TRAM2‐AS1 in modulating susceptibility to ferroptosis.

## 6. Limitations

This study has several limitations. First, the findings are based on the TCGA dataset, and future research should conduct further validation through prospective clinical cohorts or independent public datasets. Second, additional in vitro experiments are needed to elucidate the specific mechanisms through which AC027348.1 regulates ferroptosis.

## Author Contributions

Qianru Li, Aijie Zhang, and Mengyao Lv performed the experiments. Qianru Li, and Mengyao Lv wrote the paper. Xue Liu and Wanting Lu analyzed and interpreted the data. Min Shao contributed to reagents, materials, analysis tools, or data. Limian Cao contributed to reagents, materials, conceived, and designed the experiments.

## Funding

This work was funded by the National Natural Science Foundation of China Youth Program, 32100622; the Natural Science Foundation of Anhui Province, 2008085QC113; the Natural Science Research Program of Anhui Universities, 2023AH053286; the Youth Training Program of the First Affiliated Hospital of Anhui Medical University, 2020KJ25; and the First Affiliated Hospital of Anhui Medical University Doctoral Talent Training Program, BSKY2019032.

## Conflicts of Interest

The authors declare no conflicts of interest.

## Supporting Information

Supporting Table S1. Ferroptosis markers.

Supporting Table S2. Ferroptosis‐related lncRNAs.

Supporting Table S3. Ferroptosis‐related lncRNAs, which correlated with patient prognosis.

Supporting Table S4: All primer information.

Supporting Table S5. Three subtypes of GSEA enrichment analysis.

Supporting Table S6. 6441 differentially expressed genes detected by RNA‐seq analysis after AC027348.1 knockdown in OVCAR8 cells.

Supporting Table S7. 3509 Differentially expressed gene enrichment analysis.

Supporting Table S8. Ferroptosis marker, inhibitor, and suppressor.

Supporting Figure 1: Differentially expressed gene enrichment analysis.

Supporting Figure 2: Subcellular localization of AC027348.1.

Supporting Figure 3: AC027348.1 regulates ferroptosis through multiple pathways.

## Supporting information


**Supporting Information** Additional supporting information can be found online in the Supporting Information section.

## Data Availability

The data that support the findings of this study are available in the University of California Santa Cruz (UCSC) Genome Browser database at https://genome.ucsc.edu/. These data were derived from the following resources available in the public domain: UCSC Genome Browser, which provides access to a comprehensive array of genomic data across various organisms; the URL is https://genome.ucsc.edu/. As for our transcriptome sequencing for the cell experiments, it was performed by BGI, and the figures were generated directly through their website; the URL is https://biosys.bgi.com/#/report/login. Furthermore, we guarantee that all the data from our cell experiments is genuine; the original raw data pertaining to all cell experiments will be made available to the editor.
