# Supplementary material for: Molecular Subtype Classification and Mechanistic Investigation Based on Ferroptosis‐Related lncRNAs in Ovarian Cancer
Source: Genet Res (Camb). 2026 Mar 20;2026:4503115. doi: 10.1155/genr/4503115 (PMC13140908; doi:10.1155/genr/4503115)
Supplement: Supplementary file 1 — Supporting Information Additional supporting information can be found online in the Supporting Information section. [file GENR-2026-4503115-s001.zip › Description for Supplementary Figures1-3.docx]

**Supplementary Figure 1: Differentially expressed genes enrichment analysis.**

(a–c) GO enrichment analysis of 3059 differentially expressed genes associated with different biological processes (BP), molecular function (MF), and cellular components (CC).

**Supplementary Figure 2: Subcellular Localization of AC027348.1**

(a-c) Subcellular Localization of AC027348.1 in OVCAR8, OVCAR433, and SKOV3 Cell Lines.

**Supplementary Figure 3：AC027348.1 regulates ferroptosis through multiple pathways**

(a-b) AC027348.1 was knocked out in OVCAR8 and SKOV3 cell lines, respectively. The expression levels of AC027348.1 and the NRF2 gene were subsequently validated by RT-qPCR, while alterations in NRF2 protein levels were examined using Western blotting. (c) The knockout of NRF2 was verified by Western blotting. Cell viability of NRF2-KO cells following 6-hour RSL3 treatment was assessed using a CCK-8 kit. (d-e) VENN plot showing the number of shared and unique ferroptosis-related genes (green) versus the number of differentially expressed genes (blue) identified by RNA-seq analysis in NRF2-KO cells; the heatmap illustrates the differences in gene expression levels. (f)VENN plot showing the number of WT ferroptosis-related genes (green) versus the number of differentially expressed genes (blue) identified by the presence of ferroptosis-related genes in NRF2-KO cells. (g) The circular heatmap for gene expression difference analysis compares the expression changes of multiple genes in different experimental groups (WT Sh-AC027348.1 VS Ctrl and NRF2 KO Sh-AC027348.1 VS Ctrl).
