# Supplementary material for: Molecular Subtype Classification and Mechanistic Investigation Based on Ferroptosis‐Related lncRNAs in Ovarian Cancer
Source: Genet Res (Camb). 2026 Mar 20;2026:4503115. doi: 10.1155/genr/4503115 (PMC13140908; doi:10.1155/genr/4503115)
Supplement: Supplementary file 1 — Supporting Information Additional supporting information can be found online in the Supporting Information section. [file GENR-2026-4503115-s001.zip › Supplement figures.pptx]

## Slide 1
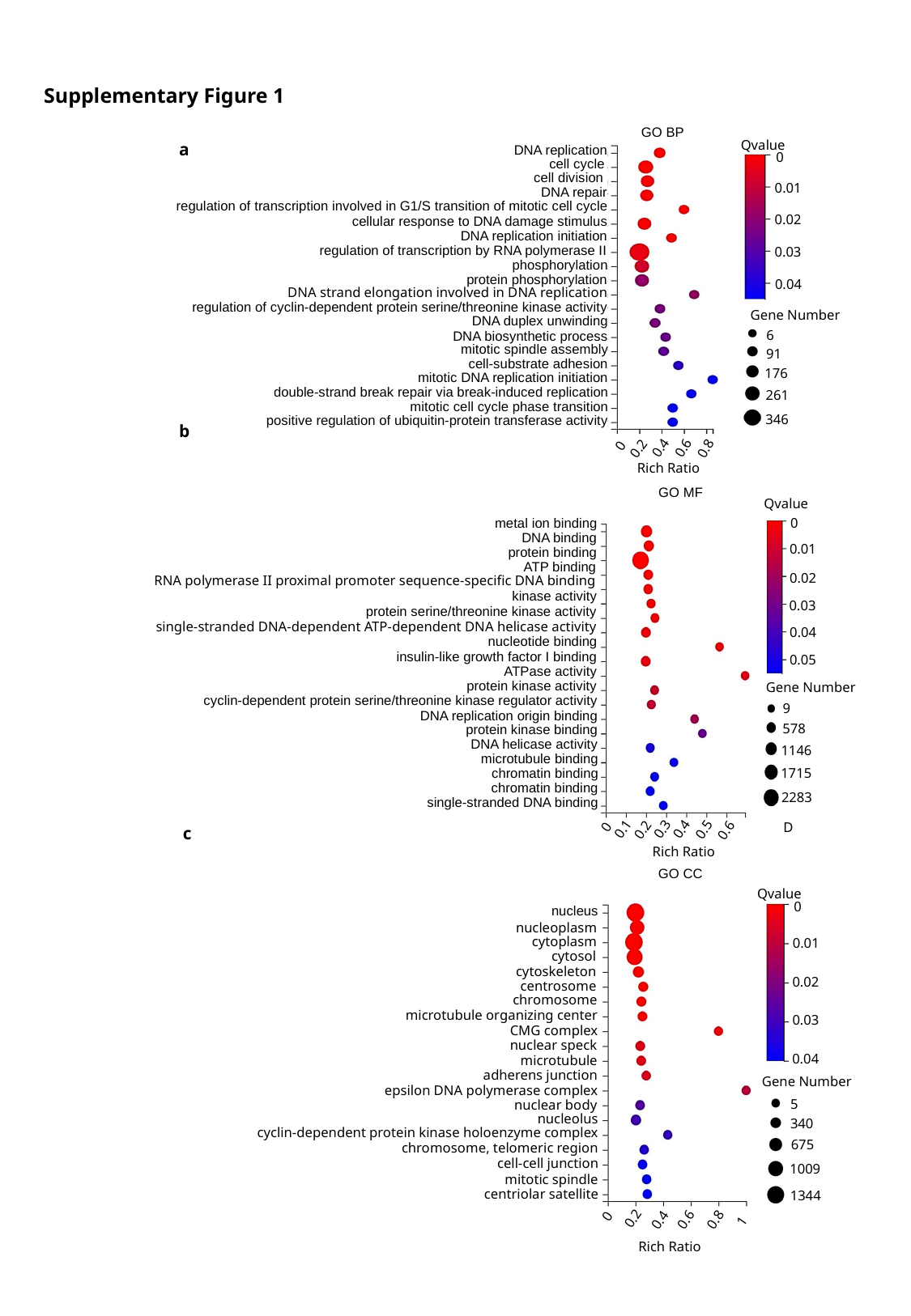

Supplementary Figure 1
GO BP
DNA replication
cell cycle
cell division
DNA repair
regulation of transcription involved in G1/S transition of mitotic cell cycle
cellular response to DNA damage stimulus
DNA replication initiation
regulation of transcription by RNA polymerase II
phosphorylation
protein phosphorylation
DNA strand elongation involved in DNA replication
regulation of cyclin-dependent protein serine/threonine kinase activity
DNA duplex unwinding
DNA biosynthetic process
mitotic spindle assembly
cell-substrate adhesion
mitotic DNA replication initiation
double-strand break repair via break-induced replication
mitotic cell cycle phase transition
positive regulation of ubiquitin-protein transferase activity
0.6
0.8
0.2
0
0.4
Rich Ratio
Qvalue
0
0.01
0.02
0.03
0.04
6
91
176
261
346
Gene Number
a
b
GO MF
metal ion binding
DNA binding
protein binding
ATP binding
RNA polymerase II proximal promoter sequence-specific DNA binding
kinase activity
protein serine/threonine kinase activity
nucleotide binding
insulin-like growth factor I binding
ATPase activity
protein kinase activity
DNA replication origin binding
protein kinase binding
DNA helicase activity
microtubule binding
chromatin binding
chromatin binding
single-stranded DNA binding
cyclin-dependent protein serine/threonine kinase regulator activity
single-stranded DNA-dependent ATP-dependent DNA helicase activity
0
0.01
0.02
0.03
0.04
0.05
9
578
1146
1715
2283
0
0.1
0.4
0.3
0.5
0.6
0.2
Rich Ratio
Qvalue
Gene Number
D
c
GO CC
nucleus
nucleoplasm
cytoplasm
cytosol
cytoskeleton
centrosome
chromosome
microtubule organizing center
CMG complex
nuclear speck
microtubule
adherens junction
epsilon DNA polymerase complex
nuclear body
nucleolus
cyclin-dependent protein kinase holoenzyme complex
chromosome, telomeric region
cell-cell junction
mitotic spindle
centriolar satellite
0
1
0.6
0.8
0.4
0.2
Rich Ratio
Qvalue
0
0.01
0.02
0.03
0.04
Gene Number
5
340
675
1009
1344

## Slide 2
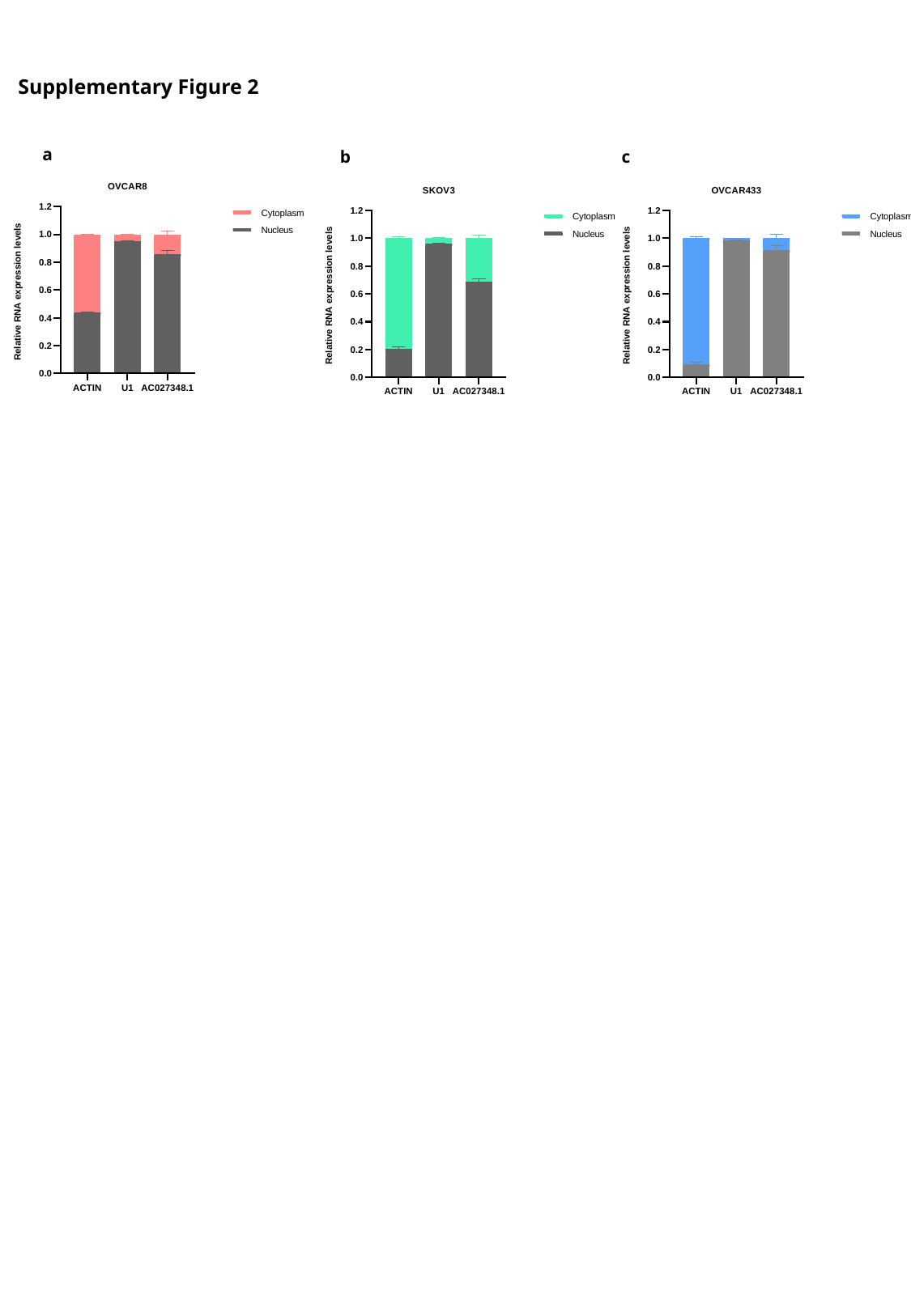

Supplementary Figure 2
a
b
c

## Slide 3
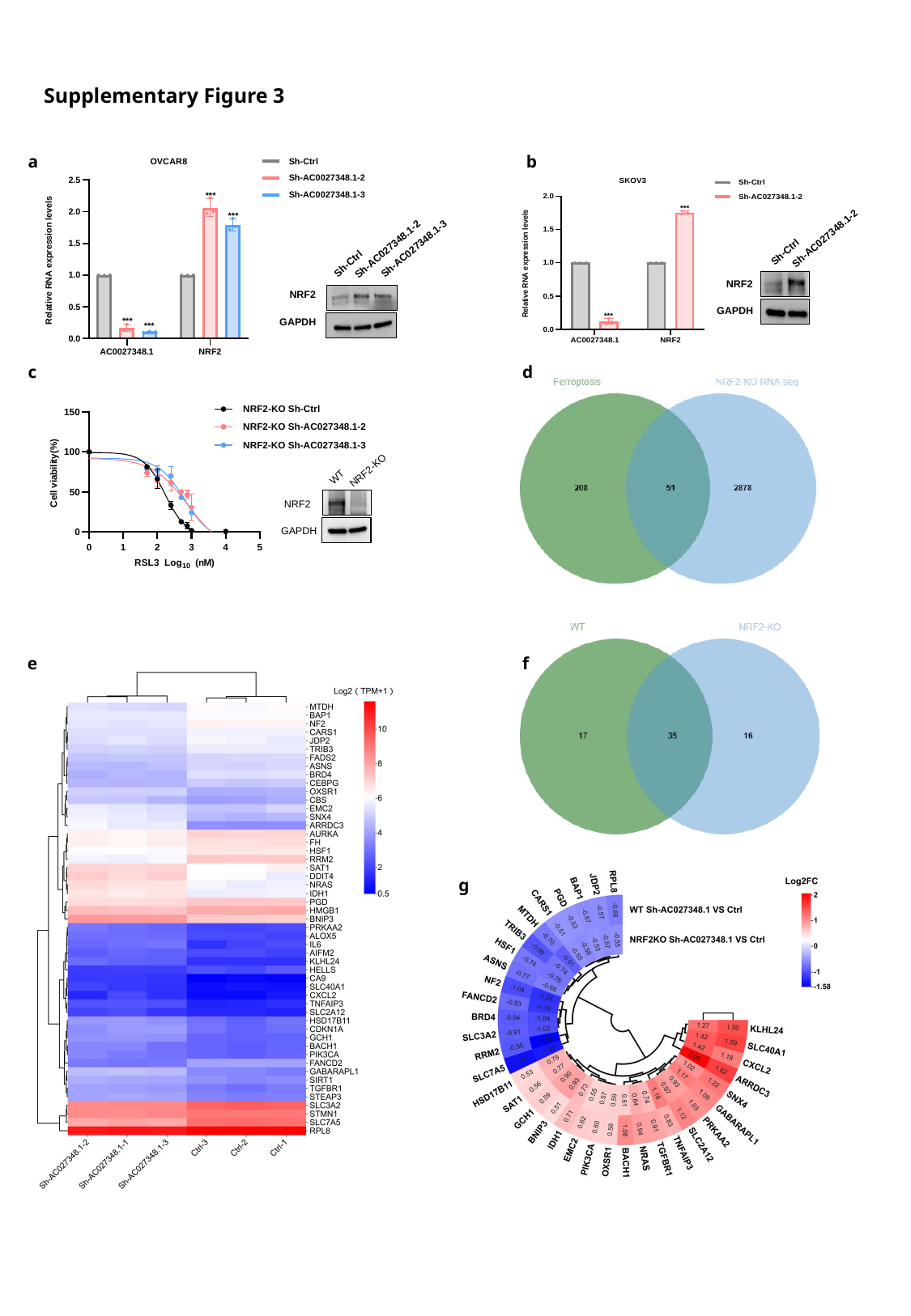

Supplementary Figure 3
a
b
Sh-AC027348.1-2
Sh-Ctrl
NRF2
GAPDH
Sh-AC027348.1-3
Sh-AC027348.1-2
Sh-Ctrl
NRF2
GAPDH
c
d
NRF2-KO
WT
NRF2
GAPDH
e
f
g
