# Supplementary material for: Molecular Subtype Classification and Mechanistic Investigation Based on Ferroptosis‐Related lncRNAs in Ovarian Cancer
Source: Genet Res (Camb). 2026 Mar 20;2026:4503115. doi: 10.1155/genr/4503115 (PMC13140908; doi:10.1155/genr/4503115)
Supplement: Supplementary file 1 — Supporting Information Additional supporting information can be found online in the Supporting Information section. [file GENR-2026-4503115-s001.zip › Supplement table s4 olige.docx]

| **shRNA sequences Sequence (5'-3')** | |
| --- | --- |
| Sh- TRAM2-AS1#1 | GCAATCACTTGCAGGGCTAAG |
| Sh- TRAM2-AS1#2 | GCTTCCCAGGAGATGCTAATG |
| Sh- TRAM2-AS1#3 | GCACTCATAGAAGTTGATTTG |
| Sh-AC027348.1#1 | GGACTTGCTGAGTGATCTGAT |
| Sh-AC027348.1#2 | GTGATCTGATGACCATTATTA |
| Sh-AC027348.1#3 | GAGGTTTATCTCTTCTGTTGA |

**Supplementary Table S4:** **All primer information.**

| **Primer used in RT-PCR** | | |
| --- | --- | --- |
| **Name** | **Sequence (5'-3')** | |
|  | **Forward** | **Reverse** |
| TRAM2-AS1 | CCAGCTGCTCTTCCTCTTCT | TGACGCCTTTAAGCACACAC |
| AC027348.1 | ACCCAGTCCTGCACACTTAG | GCAAGGCAATCAAGTGGACA |
| PART1-AS1  WAC-AS1  POLH-AS1  AC021016.1  AC007383.1  AC010336.5 | CTTCTCGTACGCTGGGCTAT  TGTGCCAGGCCTAGGTTAAA  AGAACTAAAGGATCTGCAGCC  CTAGCCTCAAAGACCCTCGT  CCTACATCTGGGGACTGCTC  CTCGACCTTGACCGCCTC | TTGTTCCAGTGCAGCCCTTT  CAAGGAGCCAAAATTGCAGC  GTAAAACTGCCCAGGGAAGC  TCAGTGTGCTTGACCCTCAT  AGGAAGATGTAGGGAGCAGC  AGAATAAAACACCGAATTGCAG |
| DYM-AS1 | TGCTGGGTGAGAAGAAAGCT | CCAAAGCTCAACCACAGTCC |
| MBTPS1DT | GCGTCGCTCTAGGATCCC | CGCCAGGCAGTCTTTCTAGA |
| AC011445.1 | AATGGCGAGGCGAGATCTAG | CACAGCAACAACTCCCGTTT |
| AL109615.3 | CACAAAAGCTCATGGCCCAG | AGGGCAACGCATGGTTAAGA |
| SLC7A11 | TGGAACGAGGAGGTGGAGAA | TGTGCTTTTTCCTTCACAGCG |
| U1 | GGCGAGGCTTATCCATTG | CCCACTACCACAAATTATGC |
| U6 | GCTTCGGCAGCACATATACTAAAAT | CGCTTCACGAATTTGCGTGTCAT |
| ACTIN | CACTGTCGAGTCGCGTCC | CGCAGCGATATCGTCATCCA |
